# Effective Combination of Photodynamic Therapy and Imiquimod 5% Cream in the Treatment of Actinic Keratoses: Three Cases

**DOI:** 10.1155/2013/102698

**Published:** 2012-12-27

**Authors:** Laura Held, Thomas Kurt Eigentler, Ulrike Leiter, Claus Garbe, Mark-Jürgen Berneburg

**Affiliations:** ^1^Department of Dermatology, Center for Dermatooncology, University Hospital Tübingen, 72076 Tübingen, Germany; ^2^Department of Dermatology, Eberhard Karls University, Liebermeisterstraße 25, 72076 Tübingen, Germany

## Abstract

*Background*. The therapy for actinic keratoses includes photodynamic therapy (PDT) and imiquimod 5% cream. The sequential use of both could result in better clinical outcomes. *Objectives*. To enhance efficacy of therapies while improving tolerability, convenience, and patient adherence with a scheme combining two concomitant or sequential AK treatments. *Methods*. All patients underwent one session of conventional PDT. Two weeks after, the PDT imiquimod 5% cream was applied to the treatment area once daily for three days per week. One course continued for four weeks followed by a clinical evaluation and decision about further treatment. Patients who had not cleared all of their AK lesions in the treatment area in course 1 participated in a second 4-week course of treatment. *Limitations*. Small size of population. *Results*. Three participants were enrolled. Two patients showed complete clinical clearance of AKs. The effect was also noted after long-term followup, at months seven and eleven. No subject discontinued for an adverse event. There were severe local skin reactions in two participants which were severe erythema, scaling, and crusting. One patient showed no response to the therapy. *Conclusions*. Photodynamic therapy followed by imiquimod was well tolerated and improved reduction of actinic keratoses. This initial proof-of-concept should be studied in larger clinical trials.

## 1. Introduction

Actinic keratoses (AKs) are common lesions which are induced by chronic sunlight exposure [1]. The lesions are frequently found on the hands, forearms, face, and scalp. A presentation of multiple AKs in a large area results in a field cancerization. AKs represent an in situ cancer and they belong to the group of nonmelanoma skin cancer (NMSC). The majority of the lesions may clear spontaneously; some lesions undergo transformation into invasive squamous cell carcinoma (SCC). The incidence of AK is increasing; the prevalence varies depending on the population. The risk factors for the development of AKs include high age, fair skin type, immunosuppression, and cumulative ultraviolet (UV) exposure. The link between the risk factors and AKs is well established and supported by a large body of epidemiological data. 

A variety of treatment strategies are available for AKs; they have specific risks and benefits and include topical agents and surgical procedures [2]. Field-directed therapies use topical agents to treat multiple AKs over a large treatment area and require from weeks to months of use. To enhance efficacy of therapies while improving tolerability, convenience, and patient adherence scheme combining two concomitant or sequential AK treatments (photodynamic therapy, imiquimod, diclofenac, cryotherapy, 5-fluorouracil, salicylic acid, and tretinoin) [3–10] has already been assessed in clinical trials.

 To further improve the duration and long-term results of the treatment, we also evaluated an alternative dosing regimen including sequential photodynamic therapy (PDT) and imiquimod. PDT is noninvasive targeted treatment which uses visible light to activate a photosensitizing agent, resulting in the formation of cytotoxic reactive oxygen species. This procedure leads to diseased tissue destruction. The therapy is highly effective in the treatment of AKs [11]. Imiquimod 5% cream regulates the level of cytokines and modifies immune response. The approved regimen in the United States for the treatment of AKs is the twice weekly use of imiquimod 5% cream for 16 weeks [12]. Imiquimod 5% cream is used as a treatment for AKs, superficial BCCs, and external genital warts. More than 95% of patients develop local skin responses such as erythema and crusting [13]. Different regimens like interval, pulse, and cycle therapies with shorter duration of treatment than conventional treatment periods have also shown to provide comparable efficacy while minimizing the local skin toxicity [14]. 

Due to different mechanisms of action, better clinical long-term results with less toxicity are expected from applying the PDT and imiquimod 5% cream sequentially. 

The aim of this investigation was to determine the efficacy, tolerability, safety, and cosmetic outcome of PDT with 5% imiquimod cream sequential treatment in patients with AKs and to give the background for the initiation of future studies in order to confirm the results and evaluate long-term therapeutic benefits. 

## 2. Patient's Selection and Methods

### 2.1. Eligibility

Patients were eligible if they were at least 18 years old and had clinically typical visible AK lesions located anywhere on the head or hands. Hyperkeratotic or hypertrophic AK lesions were not excluded. Patients were excluded if they were organ transplant recipients or if they had any dermatological disease or condition in the treatment or surrounding area.

### 2.2. Treatment Procedure

The lesions were gently abraded using a curette (Stiefel) so that the surface crust was removed. 5-aminolevulinic acid (ALA) was applied for 4 h, respectively, for AK. The cream was all the time occluded. In addition, regional anesthesia was applied for 30 min prior to irradiation. The Aktilite CL128 laser was used for irradiation in all patients. 

### 2.3. Investigation Design

Baseline screening was conducted before PDT. The lesions were measured and the exact location of AKs was documented with digital photography. Two weeks after the single session of PDT, patients started to apply imiquimod 5% cream to the treatment area once daily, three days per week. Imiquimod was left on the skin for at least eight hours before being washed off. Treatment continued for four weeks (course 1) followed by a clinical evaluation and decision about the further procedure. Patients without clinically visible AK lesions in the treatment area were considered to be completely clear and their treatment ended. Patients who had not cleared all of their AK lesions in the treatment area at the end of course 1 participated in a second four-week course of treatment. Patients were clinically evaluated at least six months after the end of the treatment—one patient withdrew from further treatment because of a lack of response to the treatment. All patients gave written informed consent to participate in this investigation. 

## 3. Case Reports

### 3.1. Case  1

**Figure 1:**
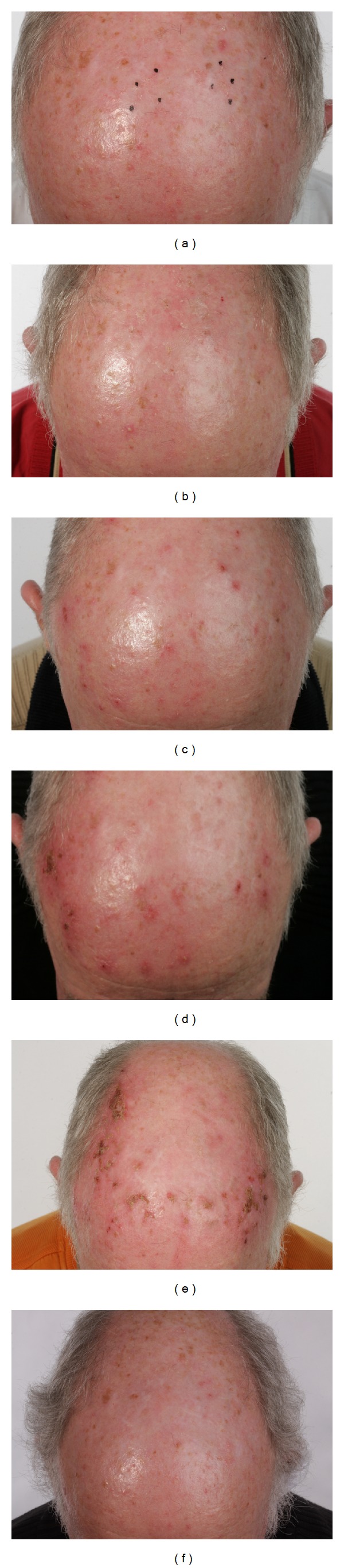
Clinical followup, Case 1. (a) Status before PDT; several hyperkeratotic lesions, hardly visible, and palpable (b) two weeks after PDT, before imiquimod; (c) two weeks after imiquimod; (d) four weeks after imiquimod, end of first course; (e) four weeks after imiquimod, end of second course; (f) seven months after the end of treatment: no visible or palpable AKs and less hyperpigmentation.

Mr. JR is male patient, 64 years old with Fitzpatrick skin phototype II. He presented with numerous hyperkeratotic lesions on his scalp (Figure 1(a)). Several actinic keratoses were clinically identified. Two well-defined target lesions were selected on the forehead for examination. After a single session of PDT (Figure 1(b)), the patient treated the target lesions and each hyperkeratotic region with an application of imiquimod three times per week for one course in accordance with the treatment protocol. The follow-up control two weeks after the imiquimod treatment showed mild erythema, erosion, and some desquamation (Figure 1(c)). These side effects were never disturbing to the patient. Four weeks after the usage of imiquimod, the local side effects (LSE) were more intense-severe erythema, scaling, and crusting. Mr. JR reported an additional development of numerous small vesicles in the treatment area. The patient felt moderately affected by the treatment (Figure 1(d)). The second course of treatment was repeated. The local side effects were intense and the patient was still moderately affected by the treatment (Figure 1(e)). The actinic keratoses healed completely after both courses of treatment and the cosmetic results were excellent. After seven months of followup, Mr. JR still had no apparent actinic lesions on his scalp or forehead (Figure 1(f)).

### 3.2. Case  2

**Figure 2:**
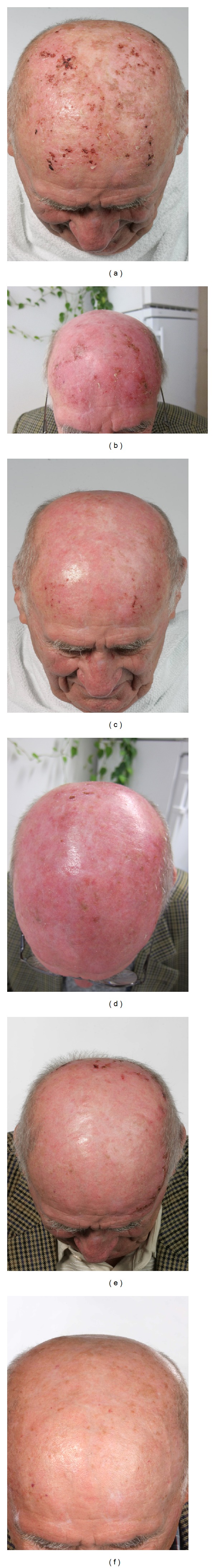
Clinical followup, Case 2. (a) Two weeks after PDT, before imiquimod; (b) two weeks after imiquimod; (c) end of first course; (d) two weeks after imiquimod, second course; (e) four weeks after imiquimod, end of second course; (f) eleven months after the end of treatment: even surface and no visible or palpable AKs.

Mr. HF is a male patient, 82 years old with Fitzpatrick skin phototype III. Numerous hyperkeratotic lesions were localized on his scalp. Two target lesions on his forehead were chosen for examination. The reaction to the PDT-treatment was pronounced (Figure 2(a)). After the healing of the wounds, imiquimod was applied to the target lesions and surrounding tissue in accordance with the treatment protocol. The patient reported mild erythema, erosion, and scaling during the follow-up control two weeks after the imiquimod treatment (Figure 2(b)). After completing the first session four weeks later, the local side effects had decreased with only moderate erythema, scaling, and crusting. The patient felt moderately affected by the treatment (Figure 2(c)). After reevaluation, the second course of treatment was repeated and this resulted in milder LSE, and the patient was hardly affected by the treatment (Figures 2(d)-2(e)). Eleven months later, Mr. HF still had no apparent actinic lesions on his scalp or forehead and he was highly satisfied with the clinical and cosmetic outcome (Figure 2(f)).

### 3.3. Case  3

**Figure 3:**
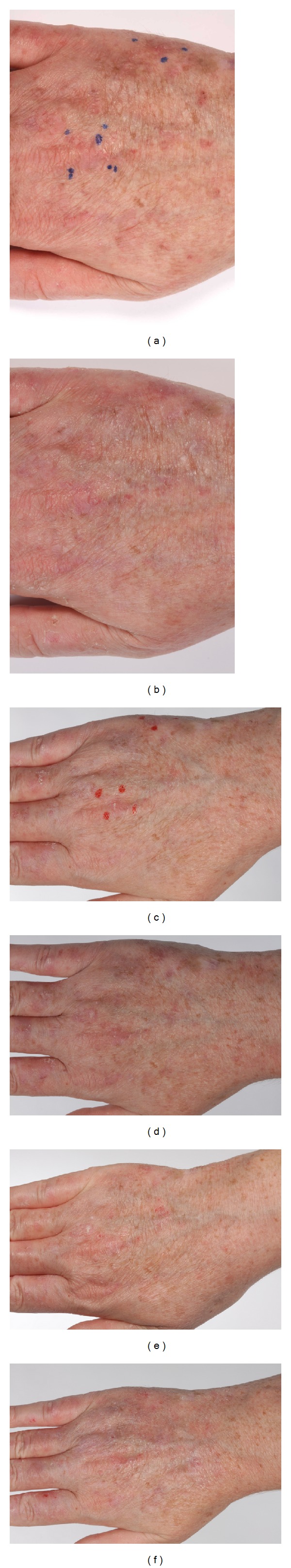
Clinical followup, Case  3. (a) Before PDT; (b) two weeks after PDT; (c) two weeks after imiquimod; (d) four weeks after imiquimod, end of first course; (e) two weeks after imiquimod, second course; (f) four weeks after imiquimod, end of second session: less scaling of the skin, more homogenous surface, the skin texture appeared even better, and AKs still present.

Mrs. AD is a female patient, 42 years old with Fitzpatrick skin phototypes II-III. Numerous hyperkeratotic lesions were localized on her hands (Figure 3(a)). Two target lesions on her right hand were chosen for examination. Following by the PDT, imiquimod was applied to the target lesions and surrounding tissue in accordance with the treatment protocol. The patient reported some erythema, edema, and scaling directly after the PDT (Figure 3(b)). But there were no local side effects noted in the follow-up control two weeks after the imiquimod treatment (Figure 3(c)). After completing the first session four weeks later, there were still no local side effects observed (Figure 3(d)). The patient was not affected at all by the treatment. Mrs. AD reported, however, subjective improvement of the skin—she noted less scaling of the skin. After reevaluation, the second course of treatment was repeated. No local side effects were noted (Figures 3(e)-3(f)). When compared to baseline photographs, the skin showed a more homogenous surface and the skin texture appeared even better. However, the actinic keratoses were still present (Figures 3(e)-3(f)). 

## 4. Discussion

The findings of this investigation demonstrate that the sequential application of PDT and imiquimod 5% cream is a well-tolerated method in the treatment of actinic keratoses with high efficacy and safety and with an excellent cosmetic outcome. Sequential application of PDT and imiquimod has not been widely evaluated in the literature. There are only two known published studies that have compared the sequential application of PDT and imiquimod 5% cream [4, 15]. Nevertheless, the regimen differs from our evaluation. Serra-Guillen et al. compared imiquimod 5% cream, single-session PDT, and the sequential application of both [15]. They showed that the sequential application of both therapeutic modalities provides a better clinical and histological response than monotherapy either with PDT or with imiquimod. It also produces less intense local reactions and better tolerance and satisfaction than imiquimod monotherapy. With 105 patients completing the study, this was a representative evaluation. Nevertheless, the study protocol differs from the protocol in the present investigation; the imiquimod cream was administered four weeks after the PDT and the patients completed only one session of imiquimod. The time until the final evaluation was very short being only one month. 

Shaffelburg designed a randomized, vehicle-controlled, split-face study to explore the safety and efficacy of photodynamic therapy followed by imiquimod [4]. Facial actinic keratoses were treated with PDT at baseline and at month one. At month two, imiquimod 5% cream was applied to one-half of the face and the vehicle to the other half, twice-times per week. The duration of imiquimod therapy was long at 16 weeks. The time until the final evaluation was long at 12 months. The method was well tolerated and improved reduction of actinic keratoses resulted.

Both studies showed good results in AK treatment. In the present investigation, two out of three patients showed in long-term followup a high benefit from this therapy modality with only one patient being a nonresponder. This could be explained by high UV-damage of the skin of the patient. The patient was very young being 42 years old, and she had reported skin problems for approximately five years. Various topical treatments (imiquimod and diclofenac in hyaluronic acid as monotherapy) were tried without any side effects nor any response in the past. This could be an explanation for the lack of the additional benefit from the combined therapy. In addition, the AKs were localized on the hands and the treatment of them seems to be more complicated. The present investigation showed that a single-session PDT and a period of eight weeks of imiquimod administration would be enough to obtain good clinical results. Nevertheless, this investigation analyzed only three cases and this was a big limitation. For confirmation of the results and evaluation of long-term therapeutic benefits, further studies are needed. 

In conclusion, two patients with long-term followup showed no relapse of AKs. Sequential therapy consisting of PDT and imiquimod 5% cream seems to provide good results in the treatment of actinic keratoses.

## Abbreviations

AKs: Actinic keratosis
BCC: Basal cell carcinoma
LSE: Local side effects
NMSC: Nonmelanoma skin cancer
PDT: Photodynamic therapy
SCC: Squamous cell carcinoma
UV: Ultraviolet.
